# Spontaneous Arrest of Sporadic Spinal Hemangioblastoma Growth after Postoperative Nodular Recurrence: Case Report

**DOI:** 10.7759/cureus.3380

**Published:** 2018-09-28

**Authors:** Adam Y Li, Alexander F Post, Jennifer B Dai, Tanvir F Choudhri

**Affiliations:** 1 Neurosurgery, The Icahn School of Medicine at Mount Sinai, New York, USA

**Keywords:** hemangioblastoma, spine, sporadic, recurrence, spontaneous arrest

## Abstract

Hemangioblastomas are rare, slow-growing, highly vascularized tumors of the central nervous system which often occur in the spinal cord. When presenting as sporadic, isolated tumors without Von-Hippel Lindau disease, they are curable through surgery with a low rate of recurrence. Tumor recurrence in these cases is usually associated with prior subtotal resection. However, to the best of our knowledge, cases of recurrent, sporadic spinal hemangioblastoma have not been reported to spontaneously arrest without intervention or symptoms. We report a patient who underwent an initial complete resection of a cervical spinal hemangioblastoma, a subtotal resection of tumor recurrence four and a half years later, and nine years of neurologic and radiographic stability with no additional interventions.

## Introduction

Hemangioblastomas are rare, slow-growing, highly vascularized tumors of the central nervous system most commonly found in the posterior cranial fossa and spinal cord [1]. The only known genetic etiology is the loss of functional Von Hippel-Lindau (VHL) tumor suppressor, in VHL disease, which is seen in 25% of hemangioblastomas. Sporadic, isolated spinal hemangioblastomas, without VHL mutation, typically present later in life as solitary lesions, cause more severe neurological deficits, and are often curable through surgery [2].

Recurrence of sporadic spinal hemangioblastomas is primarily associated with subtotal resection. To the best of our knowledge, there have been 13 described cases to date of sporadic spinal hemangioblastomas recurring postoperatively and requiring repeat surgery for symptomatic deficits [3-8]. However, no reported case of recurrent and sporadic spinal hemangioblastoma has spontaneously arrested after subtotal resection requiring no further surgical intervention. Here, the authors present the case of a 72-year-old woman who underwent two resections of recurrent cervical hemangioblastoma. Recurrence after the second operation spontaneously arrested and has remained stable for nine years.

## Case presentation

**Figure 1:**
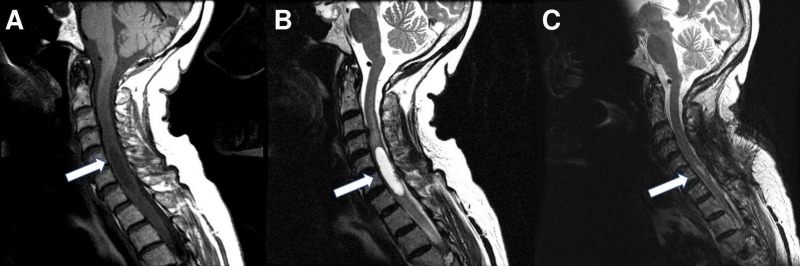
Preoperative and postoperative imaging of cervical intramedullary hemangioblastoma with associated cyst Magnetic resonance (MR) images showing a cervical syrinx and nodule on (A) preoperative sagittal T1-spin echo (SE) and (B) T2-fast recovery fast spin echo (FRFSE). (C) Postoperative sagittal T2 MR image after removal of the hemangioblastoma with decompression of the syrinx.

A 72-year-old woman presented with nine months of neck pain and left upper extremity pain and numbness, three months of right upper extremity pain, and more recent weakness in the left arm. She also had a long history of urinary stress incontinence with no changes in bowel or bladder function during the nine months of symptoms preceding her presentation. Physical examination was notable for 4/5 triceps strength, effort-limited 4+/5 left grip strength and symmetric reflexes. Magnetic resonance imaging (MRI) revealed a cervical intramedullary syrinx from C5-C7 with a nodule suggestive of a hemangioblastoma (Figure 1).

She underwent uncomplicated elective C5-C7 laminectomies and resection of the nodule. Pathological testing confirmed the lesion to be a hemangioblastoma.

**Figure 2:**
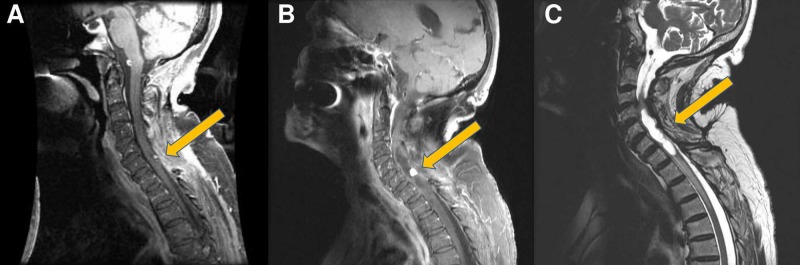
Recurrence of the hemangioblastoma and syrinx (A) Seven-month postoperative, fat-suppressed magnetic resonance imaging (MRI) with contrast shows a small enhancing lesion at C6; (B) four-year postoperative T1-fluid-attenuated inversion recovery (FLAIR) MRI with contrast; (C) MRI fast recovery fast spin echo (FRFSE) images show lesion growth with the development of a syrinx.

Imaging performed seven months postoperatively revealed abnormal hyperintensity of the left posteromedial spinal cord at C6 with a small, nodular, enhancing lesion of the most posterior portion of this abnormality. Subsequent imaging showed a progressive increase in the size of this lesion, and imaging four years postoperatively revealed increased nodular enhancement and recurrence of the syrinx (Figure 2). Four years postoperatively, she was also diagnosed with complex regional pain syndrome.

**Figure 3:**
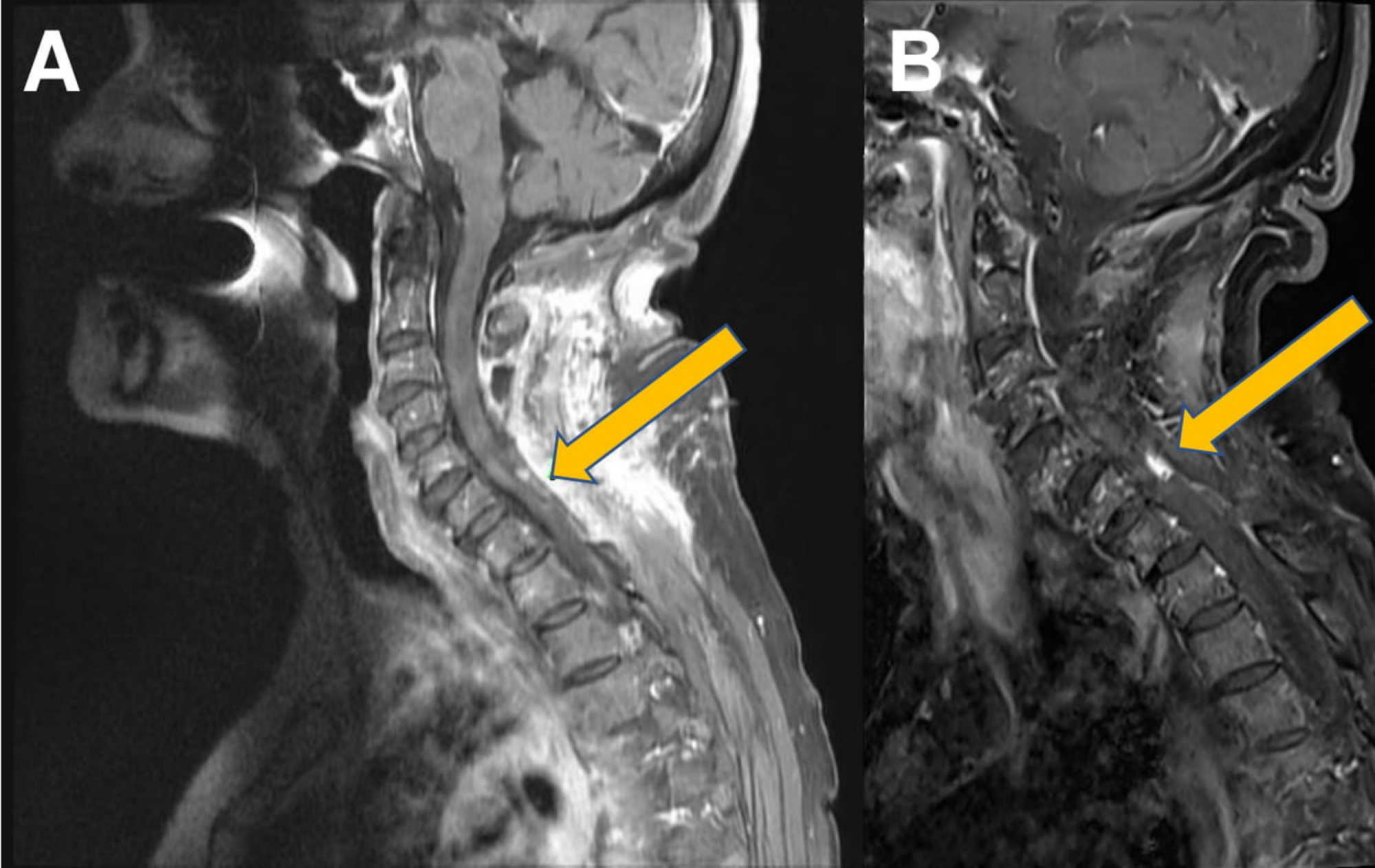
Postoperative nodule and lack of major postoperative growth (A) Fluid-attenuated inversion recovery magnetic resonance imaging (FLAIR MRI) with contrast (four and a half years after initial operation, two months after second operation); (B) FLAIR MRI with contrast (13.5 years after initial operation, nine years after second operation)

Four and a half years after her index surgery, increasing enhancement size prompted additional surgery, including extension laminotomies/laminectomy from C3-T1 and resection of the pathology-proven hemangioblastoma. Imaging two months after the second surgery showed a punctate focus of enhancement at the dorsal aspect of C6, suspicious for residual or recurrent tumor. Subsequent annual imaging, with the most recent scan nine years after the second operation, has shown no significant changes in the focus of enhancement at C6 (Figure 3). No further interventional treatment has been performed.

## Discussion

Hemangioblastomas are rare, highly vascularized tumors of the central nervous system accounting for 2 - 8% of intramedullary tumors [1]. Of patients with hemangioblastomas, the spine is the second most common site for disease, as 80% of hemangioblastoma patients develop lesions in the posterior cranial fossa and 20% of patients develop lesions in the spine [9]. Overall, understanding of the pathogenesis and role of mutated genes in the development of spinal hemangioblastomas remains limited.

One known causative factor of spinal hemangioblastomas is Von-Hippel Lindau disease caused by mutation or loss of the VHL gene. VHL encodes an E3 ubiquitin ligase that targets hypoxia-inducible factor 1α (HIF1α), which is involved in the regulation of cell metabolism and vascular proliferation [1]. Mutation of VHL is present in approximately 25% of hemangioblastoma patients [1], and 88% of hemangioblastoma patients with VHL present with spinal disease [6]. The role of VHL, as well as vascular proliferation in disease progression, makes anti-HIF1α drugs and vascular endothelial growth factor (VEGF) inhibitors, such as the angiogenesis inhibitor bevacizumab, potential treatments for spinal hemangioblastomas in the setting of VHL disease. Anti-VEGF therapies, anti-HIF1α therapies, IFN-alfa-2a, and thalidomide have shown varying levels of therapeutic value in the treatment of VHL-associated hemangioblastomas [1, 10].

Sporadic hemangioblastomas have no known causative factors. Only 20% of these lesions occur in the spinal cord, and they have a different presentation when compared to VHL-associated lesions [6]. Sporadic disease typically presents later in life (4th vs 3rd decade), leads to more severe neurological deficits, and usually presents as a single larger lesion (in contrast to smaller multiple VHL-associated lesions). Typically, they are distributed in the cervical and thoracic spine (compared to all spine levels with VHL disease), and significantly improve after surgery (while VHL lesions do not and often recur) (Table 1) [6].

**Table 1:** Common clinical characteristics of sporadic and VHL-associated hemangioblastomas VHL: Von-Hippel Lindau; HIF1α; hypoxia-inducible factor 1α; VEGF: vascular endothelial growth factor [1, 2, 6]

Types of Hemangioblastomas	Sporadic Disease	VHL-associated Disease
Prevalence of spinal lesions	20%	88%
Known genetic mutation	None	VHL tumor suppressor
Age at presentation	30-40	40-50
Neurological status at diagnosis	Mild to moderate deficits	No symptoms to mild deficits
Tumor number	Single	Multiple
Tumor size	Large	Small
Tumor distribution	Cervical and thoracic spine	All spine levels
Surgical outcome	Significant improvement	No significant improvement
Factors affecting surgical outcome	Partial removal of lesions	Partial removal of lesions
New development of lesions	6.25% - 20% recurrence rate	One-third of patients every two years
Potential new therapies	None	HIF1α and VEGF inhibitors

For both sporadic and VHL-associated hemangioblastomas, recurrence is associated with subtotal resection of lesions; however, the recurrence is rare in sporadic disease while one-third of patients with VHL-associated disease will recur every two years [6]. Overall, the rate of recurrence in sporadic disease has been reported between 6.25% and 20% [2]. Other than a VHL mutation, the clinical and molecular causes of hemangioblastoma proliferation are unknown. In our case, without VHL disease being present, it is unclear why the second recurrence spontaneously arrested after subtotal resection and no additional intervention. Interestingly, our patient was diagnosed with complex regional pain syndrome (CRPS) a year before her second operation. Various changes in genetics, inflammation signals, and circulating catecholamines have been known to affect CRPS, and it is unknown whether these factors played a role in affecting the growth of her sporadic hemangioblastoma [11]. Since sporadic spinal hemangioblastomas have been reported to recur up to 15 years postoperatively [2], it is still possible that symptomatic changes may occur. Therefore, future continued monitoring is needed. If the lesion continues to remain stable, it will indicate that subtotal resection of sporadic hemangioblastoma during surgery for recurrence does not always lead to significant growth or symptomatic disease.

## Conclusions

Recurrence of sporadic hemangioblastomas occurs less frequently than VHL-associated hemangioblastomas. When sporadic hemangioblastomas do recur, they are usually associated with a subtotal resection. To the best of our knowledge, the sporadic recurrent hemangioblastoma presented here is the first reported case to spontaneously arrest without intervention. The mechanism of spontaneous arrest is unknown. Our patient was diagnosed with complex regional pain syndrome one year before her second operation, and it is unknown whether the CRPS contributed to the arrest of the tumor.
